# Supplementary material for: The Interaction of Food Allergy and Diabetes: Food Allergy Effects on Diabetic Mice by Intestinal Barrier Destruction and Glucagon-like Peptide 1 Reduction in Jejunum
Source: Foods. 2022 Nov 22;11(23):3758. doi: 10.3390/foods11233758 (PMC9741085; doi:10.3390/foods11233758)
Supplement: Supplementary file 1 [file foods-11-03758-s001.zip › foods-1973412-supplementary.pdf]

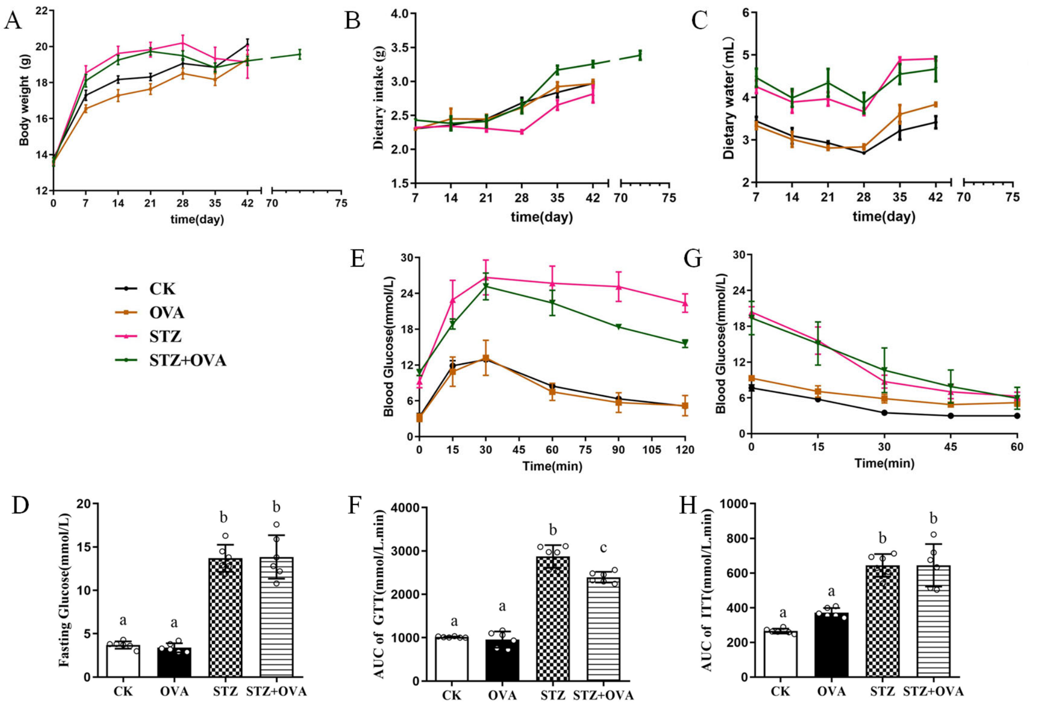

Figure S1. Computation of four groups: (A), Body weight. (B), Dietary intake. (C), Dietary water. (D), Fasting glucose. (E), Glucose tolerance. (F), AUC of glucose tolerance. (G), Insulin tolerance. (H), AUC of insulin tolerance. The error bars indicated the mean ± SEM. n = 6. Different lowercase letters in the figure represent significant differences between the groups ($p < 0.05$ by one-way ANOVA followed by SNK).

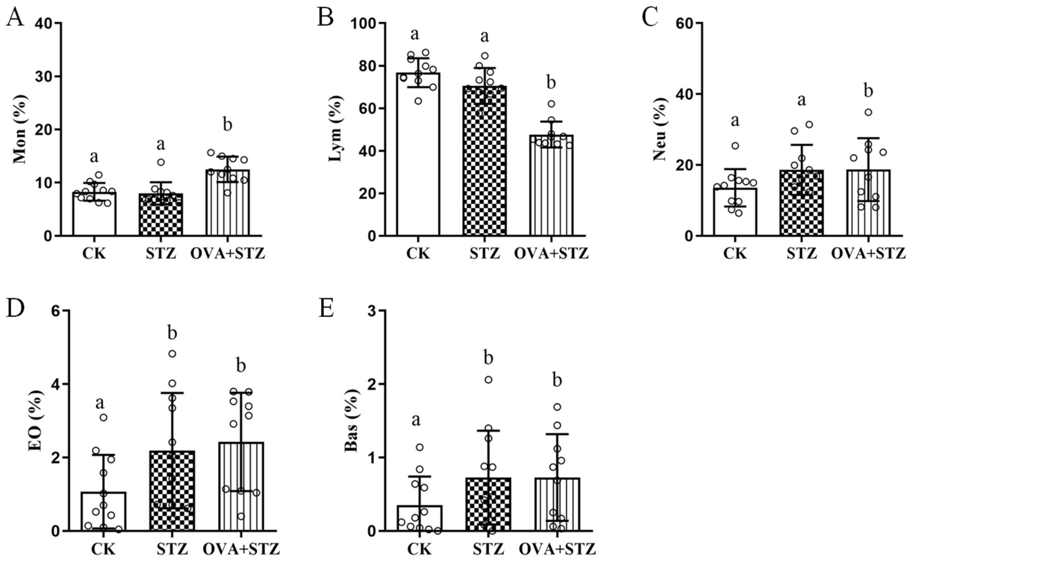

Figure S2. Leucocyte component distribution in CK, STZ, and OVA+STZ groups. (A), Mononuclear (Mon) percentage. (B), Lymphocyte (Lym) percentage. (C), Neutrophil (Neu) percentage. (D), Eosnophils (Eo) percentage. (E), Basophil (Bas) percentage. The error bars indicated the mean ± SEM. n = 6. Different lowercase letters in the figure represent significant differences

between the groups ($p < 0.05$ by one-way ANOVA followed by SNK).3.5. Food allergy affects diabetes by pro-moting jejunal barrier destruction.

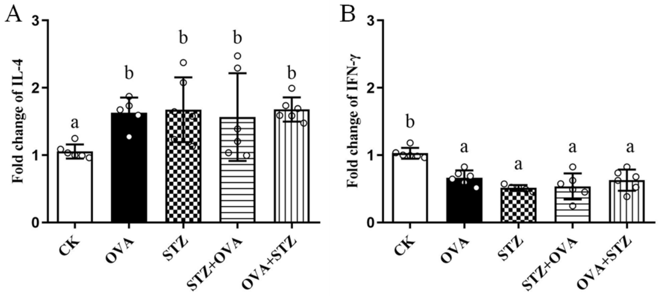

Figure S3. mRNA expression of IL-4 and IFN-γin jejunum. (A), mRNA expression of IL-4 in the jejunum. (B), mRNA expression of IFN-γ in the jejunum. The error bars indicated the mean ± SEM. n = 6. Different lowercase letters in the figure represent significant differences between the groups ($p < 0.05$ by one-way ANOVA followed by SNK).
